# Male partners’ participation in birth preparedness and complication readiness in low- and middle-income countries: a systematic review and meta-analysis

**DOI:** 10.1186/s12884-021-03994-0

**Published:** 2021-08-14

**Authors:** Minyahil Tadesse Boltena, Abraham Sahlemichael Kebede, Ziad El-Khatib, Benedict Oppong Asamoah, Andualem Tadesse Boltena, Hawult Tyae, Melese Yeshambaw Teferi, Mulatu Biru Shargie

**Affiliations:** 1 Armauer Hansen Research Institute, Ministry of Health, Addis Ababa, Ethiopia; 2 School of Health Sciences, University of Brighton, Brighton, UK; 3 World Health Programme, Université du Québec en Abitibi-Témiscamingue (UQAT), Montreal, Québec Canada; 4 Department of Global Public Health, Karolinska Institutet, Stockholm, Sweden; 5 Social Medicine and Global Health, Department of Clinical Sciences, Lund University, Lund, Sweden

**Keywords:** Birth Preparedness, Complication Readiness, LMICs, Male involvement, Participation

## Abstract

**Background:**

Maternal and neonatal health outcomes remain a challenge in low- and middle-income countries (LMICs) despite priority given to involving male partners in birth preparedness and complication readiness (BPCR). Men in LMICs often determine women’s access to and affordability of health services. This systematic review and meta-analysis determined the pooled magnitude of male partner’s participation in birth preparedness and complication readiness in LMICs.

**Methods:**

Literature published in English language from 2004 to 2019 was retrieved from Google Scholar, PubMed, CINAHL, Scopus, and EMBASE databases. The Joanna Briggs Institute’s critical appraisal tool for prevalence and incidence studies were used. A pooled statistical meta-analysis was conducted using STATA Version 14.0. The heterogeneity and publication bias were assessed using the *I*^*2*^ statistics and Egger’s test. Duval and Tweedie's nonparametric trim and fill analysis using the random-effect analysis was carried out to validate publication bias and heterogeneity. The random effect model was used to estimate the summary prevalence and the corresponding 95% confidence interval (CI) of birth preparedness and complication readiness. The review protocol has been registered in PROSPERO number CRD42019140752. The PRISMA flow chart was used to show the number of articles identified, included, and excluded with justifications described.

**Results:**

Thirty-seven studies with a total of 17, 148 participants were included. The pooled results showed that 42.4% of male partners participated in BPCR. Among the study participants, 54% reported having saved money for delivery, whereas 44% identified skilled birth attendants. 45.8% of male partners arranged transportation and 57.2% of study participants identified health facility as a place of birth. Only 16.1% of the male partners identified potential blood donors.

**Conclusions:**

A low proportion of male partners were identified to have participated in BPCR in LMICs. This calls countries in low- and middle-income setting for action to review their health care policies, to remove the barriers and promote facilitators to male partner’s involvement in BPCR. Health systems in LMICs must design and innovate scalable strategies to improve male partner’s arrangements for a potential blood donor and transportation for complications that could arise during delivery or postpartum haemorrhage.

**Supplementary Information:**

The online version contains supplementary material available at 10.1186/s12884-021-03994-0.

## Background

The low- and middle-income countries (LMICs) accounts for 84% of the world’s population and 93% of the global burden of disease [1, 2]. Maternal mortality continues to be disproportionately higher in sub-Saharan Africa (SSA), where 1 out of 39 women dies due to preventable complications of pregnancy and childbirth as compared to 1 in 3800 in Europe [3].

The 1994 International Conference on Population and Development stressed the active presence and collective responsibility of male partners in birth preparedness and complication readiness (BPCR) [4]. Engaging men in BPCR service includes informing and encouraging them to share reproductive health burdens with their wives [5–8]. This will improve women reproductive rights and behavior as significant interventions to successful maternal and child health care [9–11].

Men in LMICs are the key decision-makers on matters that influence women’s access to maternal health care services [12–22]. Affordability of basic economic needs including the majority of expenses related to essential health care services, transportation to the health facility, buying clean clothes for the baby and the mother, and arrangement of skilled pre- and post-natal care is dependent on men [2, 6, 23–29].

Additionally, nutritional requirements for both the mother and the fetus during pregnancy, and access to the postpartum emergency care depends on the out-of-pocket payment made by male partners [23, 30–32].

Studies have reported increased male partner participation in BPCR was associated with better mental health for the mother and the baby, and relief from anxiety, discomfort, and unease at the time of childbirth [20, 33–35]. Married couples in LMICs who properly practice BPCR show enhanced compliance with the use of skilled birth attendants, the prevention of mother-to-child HIV transmission program, as well as improved cognitive and socio-emotional development of children [36–41].

Furthermore, male partners involvement in BPCR is vital for improved access to prenatal and postnatal services, and discouragement of harmful maternal practices [42–44].

Sparse evidence from previous studies suggested that male partner participation in BPCR improves maternal and child health outcomes [45, 46]. However, the pooled magnitude of the association is not clear [47]. Previously conducted systematic reviews in both the developed and developing regions emphasized on the influence of male partners on non-maternal health areas such as child health outcomes and mother-to-child HIV/AIDS transmission in [40, 45, 46, 48, 49].

There is a gap in up-to-date evidence of the pooled magnitude of male partner involvement in BPCR to inform policy and impact practice in LMICs [43, 47, 50]. To fill the mentioned knowledge gap, this systematic review and meta-analysis was conducted with the aim of determining the pooled prevalence of male partner participation in BPCR in LMICs.

The review was restricted to the impact of male partners on maternal health outcomes to have a much more focused research question [51]. A preliminary search of PROSPERO [52], the Cochrane [53], and the JBI Database of Systematic Reviews and Implementation Reports [54] were conducted and no current or underway systematic reviews on the topic were identified.

## Methods

### Search strategy and selection of studies

The search strategy aimed to locate both published and unpublished literature. A preliminary search was done on Google Scholar database to identify the availability of articles on the topic. Key terms were adapted as appropriate for each database and site, with combination of MeSH terms and text words using Boolean operators “AND” and “OR” running key search topics for electronic databases such as PubMed, EMBASE, CINAHL, and Scopus (Additional file 1). The reference lists of all studies selected for critical appraisal were screened for additional studies. Both institutional and community-based cross-sectional studies published in English language from January 2004 to December 2019 were included.

Following the search, all identified citations were organized and uploaded into EndNote version 15.0 and duplicates were removed. Titles and abstracts were screened by two independent reviews and double-checked by a third reviewer for assessment against the in- and exclusion criteria. Potentially relevant studies were retrieved in full including their citation details.

Literature was eligible for inclusion if they reported the involvement of male partners of pregnant women and nursing mothers in BPCR in LMICs as participants in the study. Studies which reported the magnitude of male partners' participation in BPCR as the main outcome were included. Systematic reviews, studies conducted on women participation in BPCR, studies with poor methodological quality after a quality assessment and reports of studies conducted in high-income countries were excluded.

**Fig. 1:**
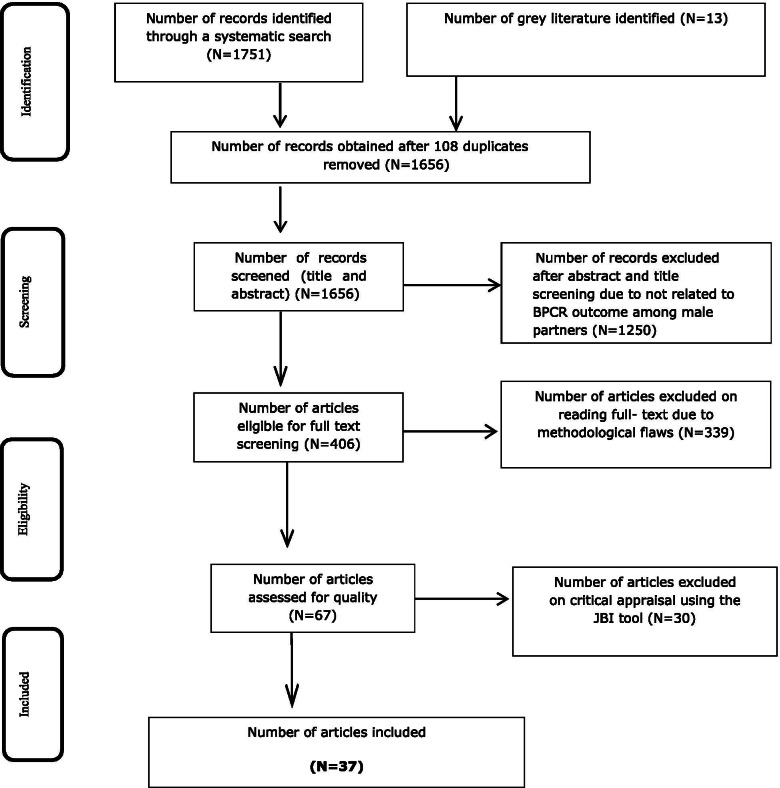
Flow diagram of the included studies. Moher, D., et al., *Preferred Reporting Items for Systematic Reviews and Meta-Analyses: The PRISMA Statement.* PLoS Medicine, 2009. 6(7)

The full text of selected citations was assessed in detail against the inclusion criteria by two reviewers and double-checked by two other independent reviewers. Reasons for exclusion of studies that did not meet the inclusion criteria up on full text screening were recorded and reported. Any disagreements that arose between the reviewers at each stage of the study selection process were resolved through discussion, or with a third reviewer. The results of the search were reported in full in the final systematic review and presented in a Preferred Reporting Items for Systematic Reviews and Meta-analyses (PRISMA) flow diagram (Fig. 1) [55].

### Operational definitions

#### Birth Preparedness and Complication Readiness

Defined as planning and organizing during pregnancy in preparation for a normal delivery or in case of complications [50, 56, 57]. The BPCR practices involves saving money for delivery; identifying transport and the location of birth of the baby; knowing danger signs of pregnancy complications [58]; identifying a skilled birth attendant and a potential blood donor [50, 56, 57]. Complications were defined as: Immediate, life threatening pregnancy or labour complications [57].

#### Birth Preparedness and Complication Readiness at a Health System Level

Is defined as a strategy of promoting the active use and retaining of well-trained human resource for maternal and neonatal health, especially during childbirth and postpartum care, based on the theory that arranging for childbirth and being prepared for complications decreases delays in receiving this care [11, 59–62].

### Male partner participation in BPCR

Refers to the knowledge, attitude, and behavioral practices associated to BPCR and emergency obstetric care by male partners of pregnant women and nursing mothers within the 42 days of the delivery of the neonate [19, 56, 63–69].

### Data extraction

The data were extracted from included studies using the data extraction tool prepared by MTB. The tool includes variables such as the name of the author, publication year, study design, data collection period, sample size, study area, and the prevalence of birth preparedness and complication readiness.

The data extraction tool contains information on the percentage of male partners who saved money for the birth of the baby, prepared a potential blood donor, identified a skilled birth attendant, and knows danger signs, arranged transportation, and identified a health facility as place of delivery of the baby. MTB extracted the data, and HT and MY cross-checked the extracted data for its validity and cleanness. Authors of papers were contacted to request missing or additional data.

### Data quality and risk of bias assessment

Eligible studies were critically appraised by two independent reviewers (MTB and MY). Methodological quality was assessed using the JBI’s standardized critical appraisal instrument for incidence and prevalence studies. The results of the critical appraisal were reported in narrative form and a table. A lower risk of bias (90%) observed after assessment (Table 1).

**Table 1:** Descriptive summary of 37 studies included in the meta-analysis of the pooled magnitude of male partners’ participation in birth preparedness and complication readiness in low- and middle-income countries, 2004 – 2020

**S. No**	**Authors**	**Year of Publication**	**Country**		**Sample size**	**Study period**	**Knowledge of danger signs**		**Saved money for delivery**		**Identified skilled birth attendance**	
							Yes	No	Yes	No	Yes	No
1	Frances Ampt	2015	Myanmar	Asia	210	July and September 2012					55 (27%)	155(63%)
2	Anna Kurniati	2017	Indonasia	Asia	1256	2012						
3	Oktaviana Betty	2019	Indonesia	Asia	504	2017						
4	Rahman et al	2018	Bangladesh	Asia	317	2015					231(73%)	86(27%)
5	Bhusa and Bhattarai	2018	Nepal	Asia	125	May–November 2016	111 (88.8)	14 (11.2)	64 (51.2)	61 (48.8)	71 (56.8)	54 (43.2)
6	Chetkant Bhusal	2015	Nepal	Asia	125	2011	52.8(66)	47.2%(59)	67.2%(84)	32.8%(41)	51.8%(64)	48.8%(61)
7	MAY CHAN OO	2019	Mynamar	Asia	198	from July to August 2018			159(80%)	39(20%)	109(55%)	89(45%)
8	Abdul-Aziz Seidu	2020	Ghana	Sub-saharan Africa	300	Jul-05						
9	Micah Matiang’i	2013	Kenya	Sub-saharan Africa	388	2010						
10	Nyasiro S. Gibore	2019	Tanzania	Sub-saharan Africa	966	June 2014 to November 2015	871 (90.2%)	95(9.8%)			227(23.5%)	739(75.5%)
11	Furaha August	2015	Tanzania	Sub-saharan Africa	756	2012	251(34.6%)	474(65.4%)	342(47.2%)	383(52.8%)	6(0.8%)	719(99.2%)
12	Richard Kalisa	2016	Rwanda	Sub-saharan Africa	327	July 2015 and November 2015						
13	Kolawole J Sodeinde	2020	Nigeria	Sub-saharan Africa	440	2016			394(89.5%)	46(10.5%)	193(43.9%)	247(56.1%)
14	Chisom J. Mbadugha	2019	Nigeria	Sub-saharan Africa	145	2017			62 (42.8%)	83(47.2%)	54 (37.2%)	91(62.8%)
15	Geoffrey C Nwakwuo	2013	Nigeria	Sub-saharan Africa	400	2011						
16	Olayinka Falade-Fatila	2020	Nigeria	Sub-saharan Africa	367	2017	362(98.6%)	5(1.4%)	79(21.5%)	288(78.5%)	318 (86.6%)	49 (13.4%)
17	Julie Osarenokemen Erhabor	2020	Nigeria	Sub-saharan Africa	372	1 Dec 2017 to 27 Jan 2018	88.2%(328)	11.8%(44)	92.7%(345)	7.3%(27)	80.4(299)	19.6%(73)
18	Ibrahim M.S	2014	Nigeria	Sub-saharan Africa	411	2012	44%(181)	230(56)	26.5%(109)	73.5%(302)		
19	Aderibigbe SA	2013	Nigeria	Sub-saharan Africa	350	2012			181(51.7%)	169(47.3%)		
20	Sisay Shine	2020	Ethiopia	Sub-saharan Africa	405	2016					359 (88.6%)	46 (11.4%)
21	Tadesse M	2017	Ethiopia	Sub-saharan Africa	608	December 2014 to January 2015	NA	NA	446(75.3%)	146(24.7)	242(40.9%)	350 (59.1)
22	Dereje Bayissa Demissie	2016	Ethiopia	Sub-saharan Africa	385	01–24 January 2015	177(47.3%)	208(52.7)	235(62.8%	150(37.2)	203(54.3%)	182(45.7)
23	Zinash Tantu	2019	Ethiopia	Sub-saharan Africa	421	March to April 2018	127(30.16%)	294(69.83%)	122(29%)	299(71%)	20(4.8%)	401(95.2%)
24	Baraki et al	2019	Ethiopia	Sub-saharan Africa	406	September 2016 to June 2017			158(39.6%)	248(60.4%)	117(29.3%)	289(70.7%)
25	Gebrehiwot et al	2013	Ethiopia	Sub-saharan Africa	398	From August to September 2012	193 (51.30)	183 (48.70)	59 (15.70)	317 (84.30)	123 (32.70)	253 (67.30)
26	Haftom G. Weldearegay	2015	Ethiopia	Sub-saharan Africa	398	From July to October/2014	193(51.30)	183(48.70)	287(76.30)	89(23.70)	123(32.70)	253(67.30)
27	Bikila Lencha Gemechu	2020	Ethiopia	Sub-saharan Africa	750	2019	402 (53.6%)	348(46.6%)	551 (73.5%)	199(26.5%)	545(72.7%)	205(27.3%)
28	Gize et al	2019	Ethiopia	Sub-saharan Africa	523	May 2016 to July 2016			159 (30.4%)	364(69.6%)	153(29.3%)	370(70.7%)
29	Bedru Hussen Mohammed	2019	Ethiopia	Sub-saharan Africa	210	2014			173(82.4%)	37(17.6%)	73(34.8%)	137(65.2%)
30	Amanual Getnet Mersha	2018	Ethiopia	Sub-saharan Africa	824	May to July 2016			218(26.5%)	606(73.6%)	67(8.1%)	757(91.9%)
31	Girma Teferi	2020	Ethiopia	Sub-saharan Africa	593	April 8 to 28 2019	184(32.06%)	390(67.94)	344(59.9%)	230(39.1%)	106(37.3%)	468(62.7%)
32	Lelise Melkamu	2019	Ethiopia	Sub-saharan Africa	362	2014	72(20.2%)	283(79.8%)	212(59.8%)	143(39.2%)	333(93.8%)	22(6.2%)
33	Melkamu Worku	2020	Ethiopia	Sub-saharan Africa	806	July 1st—30th, 2014	429(53.4%)	374(46.6%)			115(14.3%)	688(85.7%)
34	Kebreab Paulos	2020	Ethiopia	Sub-saharan Africa	233	2017					219(94%)	14(6%)
35	Aminu Mohammed	2020	Ethiopia	Sub-saharan Africa	611	Mar-20	346 (56.6%)	265 (43.4%)				
36	Fikreselassie Getachew	2019	Ethiopia	Sub-saharan Africa	422	2017	193(49%)	200(51%)	220(56%)	173(44%)	130(33%)	263(67%)
37	Alemu Tamiso Debiso	2014	Ethiopia	Sub-saharan Africa	836	April to May 2014	42%(351)	58%(485)				

**S. No**	**Identified transportation for delivery/emergency complication**		**Accompanied wife to** **antenatal care**		**Identified blood donor**		**Identified health facility as place of delivery**		**Prepared clean clothes for the baby and the mother**		**Male partner participation in BPCR (95% CI)**
	Yes	No	Yes	No	Yes	No	Yes	No	Yes	No	
1			166 (82%)	18%(44)			177 (87%)	33(13%)			134(64%)
2											1080(86%)
3											46.80%
4	67(21%)	250(79%)			38(12)	279(88%)					238(75%)
5	57 (45.6)	68 (54.4)	86 (68.8)	39 (31.2)	33 (26.4)	92 (73.6)	80 (64.0)	45 (36.0)	57 (45.6)	68 (54.4)	72(57.6%)
6	16.65%(21)	83.35(104)	48%(60)	52%(65)	12%(15)	110(88%)	69.6%(87)	38(30.4)			49.6%(62)
7	93(47%)	105(43%)			67(34%)	131(66%)	123(62%)	75(38%)			84(42.4%)
8											70%(210)
9											40%(155)
10			612(63.4%)	354(35.6%)			854 (85%)	112(15%)	746(77.3%)	220(22.7%)	521(53.9%)
11	74(10.2%)	651(89.8%)	87 (12%)	638(88%)			13 (1.8%)	712(98.2%)	394(54.3%)	331(45.7%)	87(12%)
12			78 (22.3%)	249(76.7%)							103( 29.4%)
13	387(88%)	53(12%)	177(40.2%)	263(59.8%)			300(68.2%)	140(31.8%)	422(95.9%)	18(4.1%)	232(52.7%)
14	58 (40%)	87(60%)	56 (38.6%)	89(61.4%)	23 (15.9%)	122(84.1%)	37 (25.5%)	108(74.5%)	70 (40.3%)	75(59.7%)	57 (39%)
15			275 (71.2%)	111 (28.8)							214(55.4%)
16	228 (62.1%)	139 (37.9%)	92 (25.0%)	275(75%)			218(59.4%)	149(39.6%)	339 (92.4%)	28 (7.6%)	231(63%)
17			60.7%(226)	39.3(146)					79.3%(295)	20.7%(77)	27.2%(101)
18	28.6%(111)	71.4%(287)	34%(140)	66%(271)	2.6%(10)	97.3%(401)					6.6%(27)
19			194(55.4%)	156(44.6%)			202(57.7%)	148(42.3%)	182(52%)	168(48%)	197(56.3%)
20			252 (62.2%)	153(37.8%)					359 (88.6%)	46 (11.4%)	252(62.5%)
21	357 (60.3)	235 (39.7)	301 (50.8)	291 (49.2)	108 (18.2)	484 (81.8)	437 (73.8)	155 (26.2)			266(45%)
22	235(62.8%)	150(37.2)	252(67.4%)	133(32.6)	46(12.3%)	339(87.7)	227 (60.7)	158(39.3)	239(63.9)	135(36.1)	190(50.8%)
23	102(24.2%)	319(75.8)			7(1.7%)	414(98.3%)	174(41.3%)	247(58.7%)			127(30.2%)
24	178(44.6%)	228(55.4%)	159(39.8%)	247(60.2%)	190(47.6%)	216(52.4%)	218(54.6%)	188(45.4%)	345(86.5%)	61(13.5%)	190(46.9%)
25	50 (13.30)	326 (86.70)	93 (24.70%)	283 (75.30)	65 (17.30)	311 (82.70)	234 (62.20)	142 (37.80)	301 (80.10)	75 (19.90)	227(60.4%)
26	246(65.40)	130(34.60)			65(17.30)	311(82.70)	234(62.20)	142(37.80)	301(80.10)	75(19.90)	227(60%)
27	536 (71.5%)	214(28.5%)	407 (54.3%)	343(45.7%)	133 (17.7%)	617(82.3%)	542 (72.3%)	208(27.7%)			571 (76.1%)
28	333(63.7%)	190(36.3%)			82(15.7%)	441(84.3%)	340(65%)	183(35%)	345(66%)	178(34%)	176(33.7%)
29			108(51.4%)	102(48.6%)			129(61.4%)	81(39.6%)			73(34.8%)
30	91(11%)	733(89%)	82(9.9%)	742(89.1%)	3(0.4%)	821(99.6%)	25(3%)	799(97%)	309(37.5%)	515(62.5%)	271(32.9%)
31	404(70.4%)	170(29.6%)	252(43.9%)	322(56.1%)			349(64.2%)	225(35.8%)	107(18.6%)	467(81.4%)	128(22.3%)
32			141(39.6%)	214(59.4%)							212(59.7%)
33			115(14.3%)	688(85.7%)							413(51.4%)
34	189 (81.1%)	44(18.9%)	75 (32.2%)	158 (67.8%)			223 (95.7%)	10 (4.3%)	214 (91.8%)	19 (8.2%)	72(30.9%)
35			320 (52.4%)	291 (47.6%)							40.1%(245)
36	200(51%)	193(49%)	348(88.5%)	45(11.5%)			144(36.6%)	249(63.4%)			240(61%)
37											353(42.2%)

Studies with inadequate sample size, inappropriate sampling frame and poor data analysis were excluded. Articles were reviewed using titles, abstracts, and full text screening. Full texts of included studies were examined using the Joanna Briggs Institute Meta-Analysis of Statistics Assessment and Review Instrument (JBI-MAStARI) for critical appraisal tool (Table 1).

### Data analysis

Included studies were pooled in a statistical meta-analysis using STATA version 14.0. Effect sizes were expressed as a proportion with 95% confidence intervals around the summary estimate. Heterogeneity was assessed using the standard chi-square *I*^*2*^ test. A random-effects model using the double arcsine transformation approach was used.

**Fig. 2:**
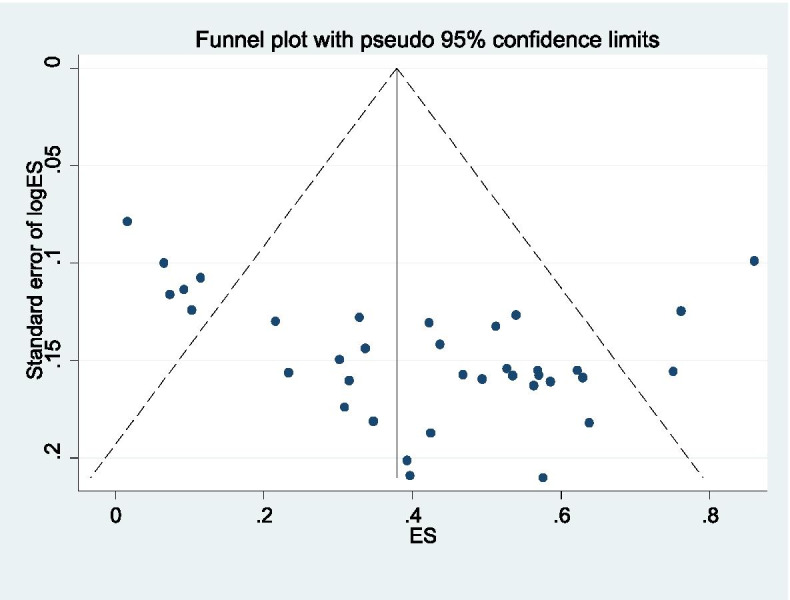
Funnel plot used to assess possible publication bias of studies published from 2004 to 2020

Sub-group analyses were conducted to investigate the level of male partner participation in the SSA and Asian regions. Sensitivity analyses were conducted to test decisions made regarding the included studies. Visual examination of funnel plot asymmetry (Fig. 2) and Egger’s regression tests were used to check for publication bias [70]. A Forest plot with 95% CI was computed to estimate the pooled magnitude of male partners’ participation in birth preparedness and complication readiness in LMICs.

### Protocol registration

The review protocol has been registered in PROSPERO with protocol registration number CRD42019140752 [71].

## Results

### Search

After removing 108 duplicates, a total of 1751 articles were obtained from MEDLINE/PUBMED, CINAHL, EMBASE, Google Scholar, and SCOPUS databases. At the title/abstract screening phase (n=1250) and during the full-article screening (n=434) articles were excluded. Accordingly, sixty-seven studies were found eligible for quality assessment. Finally, 37 studies were included in this meta-analysis (Fig. 1)

### Study characteristics

The total sample size of this systematic review was 17, 148, ranging from 125 in Nepal [19] to 1256 in Indonesia [72]. Seven studies were from Asia [19, 31, 72–76], thirty studies were from Sub-Saharan Africa [5, 22, 65, 68, 77–102]. The review was conducted on the cross-sectional study designs (Table 2).

**Table 2:** The quality assessment of 37 studies included for the pooled estimate of male partners’ participation in birth preparedness and complication readiness in low- and middle-income countries, 2004 – 2020

		**JBI quality assessmentcriteria probing questions (Q)**												
											**Study level bias score**			
**S. No**	**Included studies**	**Q-1**	**Q-2**	**Q-3**	**Q-4**	**Q-5**	**Q-6**	**Q-7**	**Q-8**	**Q-9**	**Total NoYes (Y)**	**Percentage of Yes (Y)**	**Judgment**	
**1**	Frances Ampt	Y	Y	Y	Y	Y	Y	Y	Y	Y	9	100%	Low	
**2**	Anna Kurniati	Y	Y	Y	Y	Y	Y	Y	Y	U	8	88.90%	Low	
**3**	Oktaviana Betty	Y	Y	Y	N	Y	Y	Y	Y	Y	8	100%	Low	
**4**	Rahman et al	Y	Y	Y	Y	Y	Y	Y	Y	Y	9	100%	Low	
**5**	Bhusa and Bhattarai	N	Y	Y	Y	Y	Y	Y	Y	Y	8	88.90%	Low	
**6**	Chetkant Bhusal	Y	Y	Y	Y	Y	Y	Y	Y	Y	9	100%	Low	
**7**	MAY CHAN OO	Y	Y	Y	Y	Y	U	Y	Y	Y	8	88.90%	Low	
**8**	Abdul-Aziz Seidu	N	Y	U	Y	Y	Y	Y	Y	Y	7	77.80%	Moderate	
**9**	Micah Matiang’i	Y	Y	Y	Y	Y	Y	Y	Y	U	8	88.90%	Low	
**10**	Nyasiro S. Gibore	Y	Y	Y	Y	Y	Y	Y	Y	Y	9	100%	Low	
**11**	Furaha August	N	Y	Y	Y	Y	Y	Y	Y	Y	8	88.90%	Low	
**12**	Richard Kalisa	Y	Y	Y	Y	Y	Y	Y	Y	U	8	88.90%	Low	
**13**	Kolawole J Sodeinde	Y	Y	Y	Y	Y	Y	Y	Y	N	8	88.90%	Low	
**14**	Chisom J. Mbadugha	Y	Y	Y	Y	Y	Y	Y	Y	Y	9	100%	Low	
**15**	Geoffrey C Nwakwuo	Y	Y	Y	Y	Y	Y	Y	Y	Y	9	100%	Low	
**16**	Olayinka Falade-Fatila	Y	Y	Y	Y	Y	Y	Y	U	Y	8	88.90%	Low	
**17**	Julie Osarenokemen Erhabor	Y	Y	Y	Y	U	Y	Y	Y	Y	8	88.90%	Low	
**18**	Ibrahim M.S	Y	Y	N	Y	Y	U	Y	Y	Y	7	77.80%	Moderate	
**19**	Aderibigbe SA	Y	Y	Y	Y	N	U	Y	Y	Y	7	77.80%	Moderate	
**20**	Sisay Shine	Y	Y	Y	Y	Y	Y	Y	Y	Y	9	100%	Low	
**21**	Tadesse M	Y	Y	Y	Y	Y	Y	Y	Y	Y	9	100%	Low	
**22**	Dereje Bayissa Demissie	Y	Y	Y	Y	Y	Y	Y	Y	Y	9	100%	Low	
**23**	Zinash Tantu	Y	Y	Y	Y	Y	Y	Y	Y	Y	9	100%	Low	
**24**	Baraki et al	Y	Y	Y	Y	Y	Y	Y	Y	Y	9	100%	Low	
**25**	Gebrehiwot et al	Y	Y	Y	Y	Y	Y	Y	Y	Y	9	100%	Low	
**26**	Haftom G. Weldearegay	Y	Y	Y	Y	Y	Y	Y	Y	y	9	100%	Low	
**27**	Bikila Lencha Gemechu	Y	Y	Y	Y	Y	U	Y	Y	Y	8	88.90%	Low	
**28**	Gize et al	Y	Y	Y	Y	Y	Y	Y	Y	Y	9	100%	Low	
**29**	Bedru Hussen Mohammed	Y	Y	Y	Y	Y	Y	Y	Y	Y	9	100%	Low	
**30**	Amanual Getnet Mersha	Y	Y	Y	Y	Y	Y	Y	Y	Y	9	100%	Low	
**31**	Girma Teferi	Y	Y	Y	Y	Y	Y	Y	Y	Y	9	100%	Low	
**32**	Lelise Melkamu	Y	Y	N	Y	Y	Y	Y	U	Y	7	77.80%	Moderate	
**33**	Melkamu Worku	Y	Y	Y	Y	Y	Y	Y	Y	Y	9	100%	Low	
**34**	Kebreab Paulos	Y	Y	N	Y	Y	U	Y	Y	Y	7	77.80%	Moderate	
**35**	Aminu Mohammed	Y	Y	Y	Y	Y	Y	Y	Y	Y	9	100%	Low	
**36**	Fikreselassie Getachew	Y	Y	Y	Y	Y	Y	Y	Y	Y	9	100%	Low	
**37**	Alemu Tamiso Debiso	Y	Y	Y	Y	Y	Y	Y	Y	Y	9	100%	Low	

Subtotal Yes (Y) 90%

Subtotal No (N) 3.5%

Subtotal Unclear (U) 6.5%

Overall risk of bias assessment score 90%

Remark: The risk of bias for each eligible study is calculated from the domain of nice criteria

### Pooled prevalence of birth preparedness and complication readiness

**Fig. 3:**
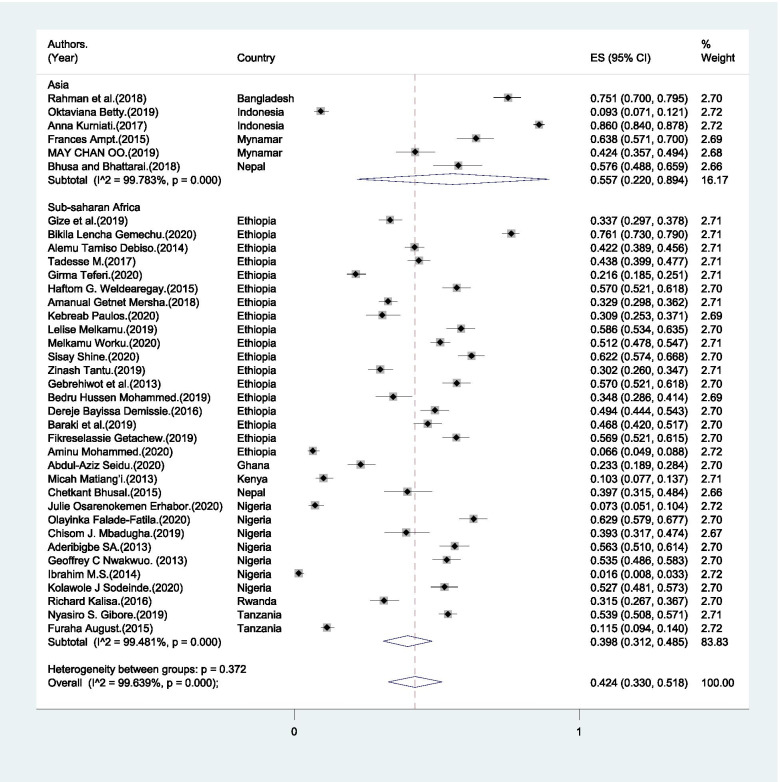
Pooled magnitude of male partners’ participation in birth preparedness and complication readiness in LMICs, 2004–2020

The range of BPCR practice among male partners was from 6.6% to 86% (Table 2). The pooled magnitude of male partner’s participation in BPCR was 42.4% (95%CI: 33.0% - 51.8%) (Fig. 3).

**Fig. 4:**
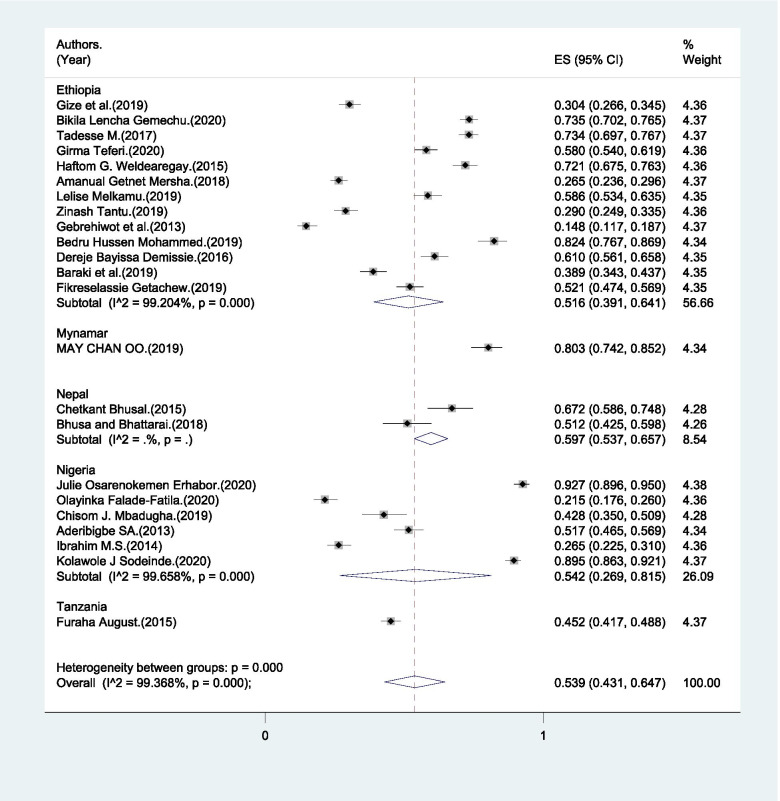
Pooled estimate of male partners’ who saved money for delivery in LMICs, 2004–2020

Saving money for delivery was varied significantly with the lowest 15.7% and the highest 92.7% (Table 2). The pooled estimate of saving money for delivery was 45.7% (95%CI: 36.7% - 54.8%) (Fig. 4). The *I*^*2*^ test result showed high heterogeneity (*I*^*2*^ = 99.27%, p= < 0.001) and Egger’s test showed no publication bias.

**Fig. 5:**
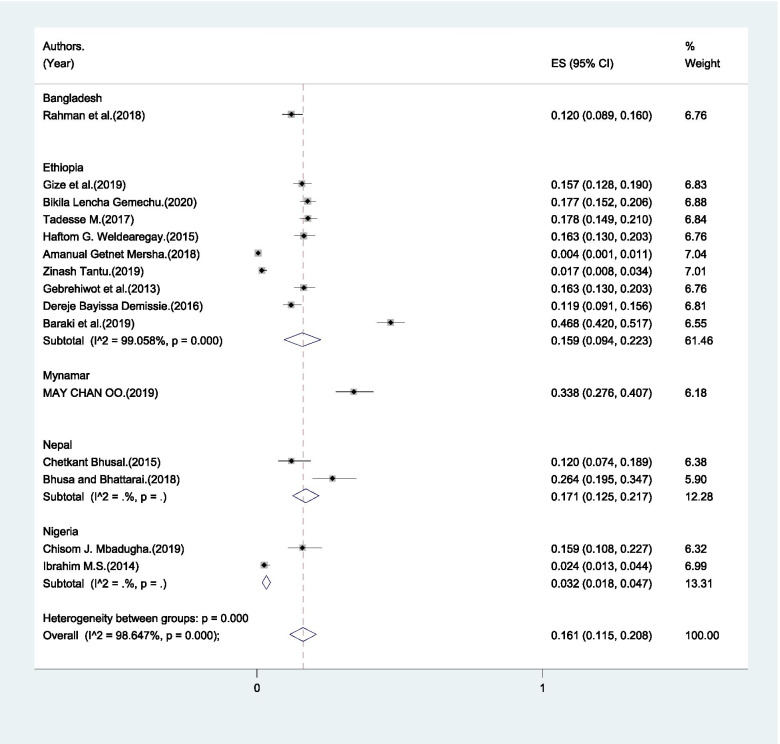
Pooled estimate of male partners’ who arranged blood donor for complications during delivery and postpartum period in LMICs, 2004-

Only 16.1% (95% CI: 11.5% - 20.8%) of male partners in LMICs were reported to have identified a potential blood donor for an emergency case that could occur during pregnancy or childbirth (Fig. 5). The minimum level of arrangement of potential blood donor was 0.4% and the maximum level was 47.6% (Table 1).

**Fig. 6:**
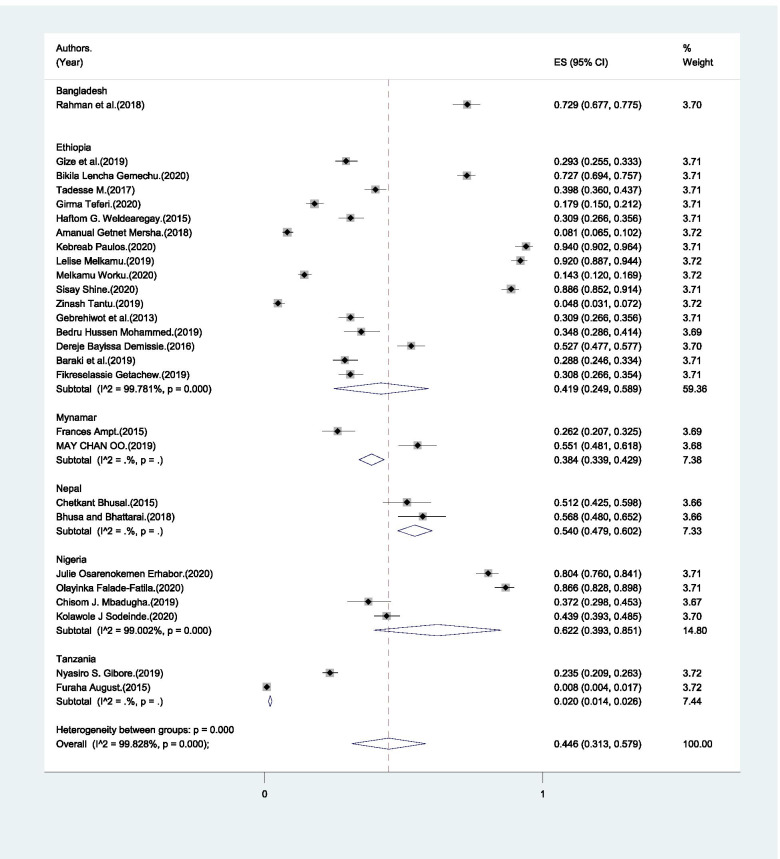
Pooled estimate of male partners’ who identified skilled birth attendant in LMICs, 2004–2020

The proportion of male partners who identified a skilled birth attendant ranged from 0.8% to 94% (Table 1). The pooled estimate of identifying skilled birth attendant was 44.6% (95% CI: 31.3% - 57.9%) (Fig. 6).

**Fig. 7:**
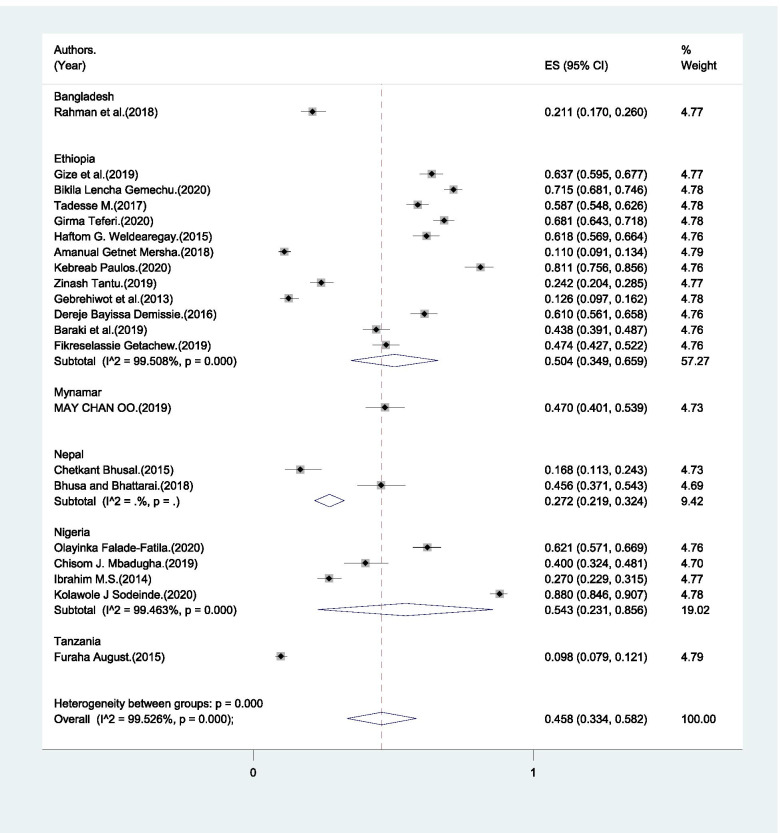
Pooled estimate of male partners’ who arranged transportation for the pregnant mother and postpartum women in global south, 2004–2020

Only 45.8% (95% CI: 33.4% - 58.2%) of male partners made transportation arrangement (Fig. 7). Arrangement of transportation by the male partners ranged from 10.2% to 88% (Table 1).

**Fig. 8:**
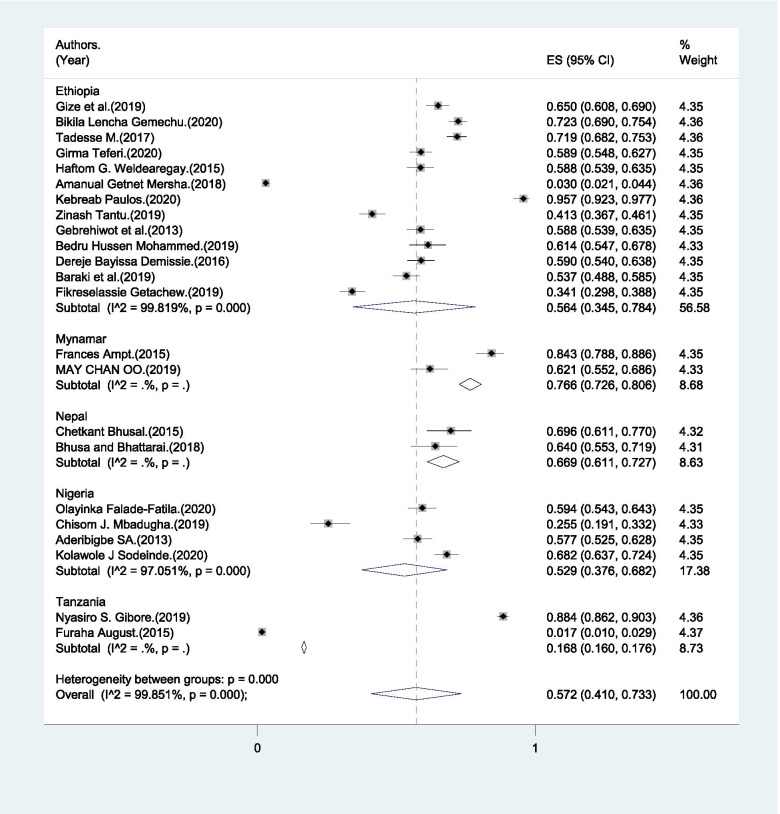
Pooled estimate of male partners’ who identified health facility as place of birth for the baby in global south, 2004–2020

A pooled estimate of 57.2% (95% CI: 41% - 73.3%) of male partners identified health facility as a place of birth for their baby (Fig. 8). Identifying health facility ranges from 1.8% to 95.7% (Table 1).

**Fig. 9:**
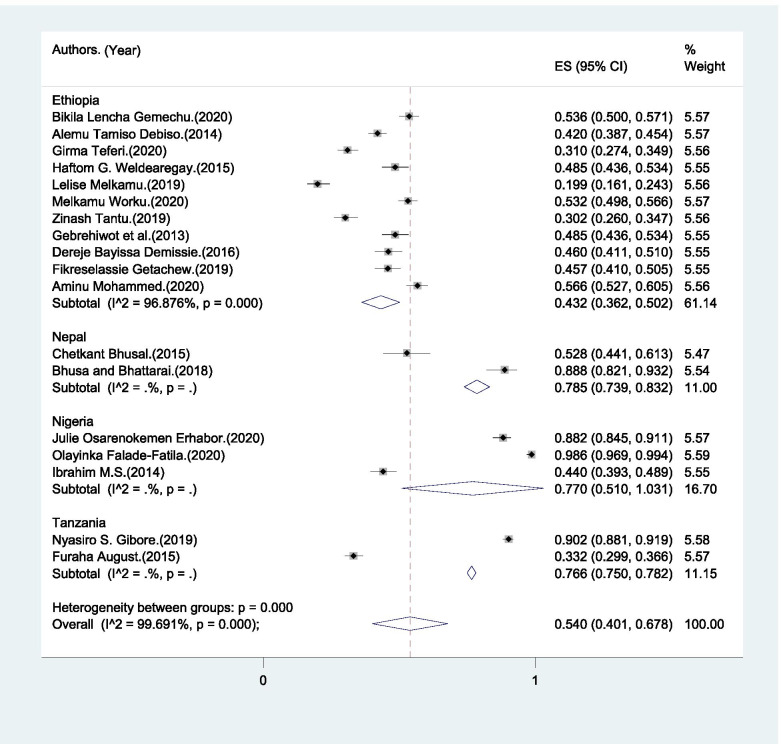
Pooled estimate of male partners’ who knew danger signs during pregnancy and childbirth in LMICs, 2004–2020

Knowledge of the danger signs that occur during pregnancy and postpartum complications was 54% (95% CI: 40.1% - 67.8%) (Fig. 9). The study that showed the least proportion of male partners with knowledge of danger sign was 20% whereas the highest was 98.6% (Table 1).

**Fig. 10:**
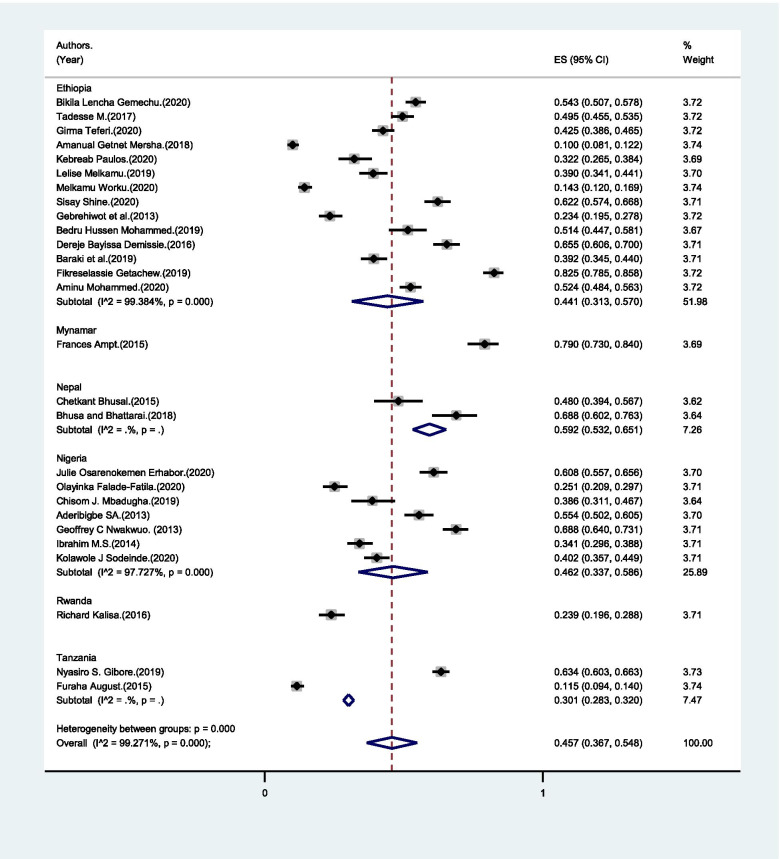
Pooled estimate of male partners’ who accompanied their wife to antenatal clinic in global south, 2004–2020

A pooled estimate of 45.7% (95% CI: 36.7% - 54.8%) of male partners accompanied their wife/partner to antenatal care follow-up (Fig. 10). The proportion of men who had antenatal clinic follow-up together with their wife/partner was reported between 9.9% and 88.5% in the different studies (Table 1).

In the sub-group analysis, the heterogeneity test indicated the presence of heterogeneity (*I*^*2*^ = 94.4%, p<0.001) but no publication bias (Egger’s test p-value < 0.001). Therefore, the pooled estimate of male partner involvement in BPCR was found to be 39.8% (95% CI: 31.2% - 48.5%) in SSA and 55.7% (95% CI: 22% - 89.4%) in Asia (Fig. 3).

## Discussion

In this review, we aimed to determine the pooled magnitude of male partner’s participation in birth preparedness and complication readiness in LMICs. Thirty-seven studies were eligible for inclusion in the meta-analysis. Only 44.6% of male partners in LMICs participated in BPCR.

The slow decline in maternal and neonatal mortality could be attributed to the underutilization of BPCR service among male partners in LMICs [21]. Poor financial readiness to pay for emergency cases during delivery and postpartum period significantly creates delayed access to emergency obstetrics and newborn care (EmONC) [103–106].

A wide range of male partner’s participation in identifying SBA was reported from SSA; The lowest proportion was among men in Tanzania, where <1% of men sought midwives care 0.8% [99], versus 94% of men, in Ethiopia, where the study participants had active involvement of identifying SBA [92]. The pooled estimate indicated that less than half of male partners (44.6%) in LMICs identified SBA. Failure to identify SBA by male partners of pregnant women and nursing mother, is among the main contributors to the disproportionate pregnancy-related complications in LMICs [69, 107].

Male partner’s financial readiness for costs related to delivery of the baby varied significantly in LMICs with the lowest (15.7%) reported from Ethiopia [84] and the highest (92.7%) indicated in Nigeria [5]. The pooled estimate of saving money for delivery was 45.7%. Only 16.1% of male partners in LMICs identified a potential blood donor for an emergency case that could occur during pregnancy or childbirth. Both the minimum and maximum levels of arrangement for a potential blood donor (0.4% and 47.6% respectively) were reported from Ethiopia [68, 83].

Postpartum hemorrhage is the leading cause of maternal mortality and it can significantly be curbed by effective enrollment and retaining of male blood donors for readily available supply of compatible blood for women who develop complications related to pregnancy and childbirth [16, 17, 104, 105, 108–110]. Compared with women, male donors are less likely to be medically late or experience vasovagal responses and are typically preferred for blood donation in voluntary settings [15, 111, 112].

The distance from the male partner’s home to a health facility and shortage of transportation during postpartum emergencies are among the barriers for the delay in reaching a health facility [16, 65, 113]. Only 45.8% of male partners in LMICs arranged for transportation to take the pregnant women and nursing mothers to delivery and post-partum complications care.

The proportion of male partners who knew the danger signs that occur during pregnancy and postpartum complications in LMICs was 54%. The study populations with both the lowest and highest levels of knowledge of danger signs of pregnancy and delivery cases were registered in SSA [114, 115]. Poor knowledge of danger signs of pregnancy and childbirth was reported from Ethiopia (20%) [90] and better knowledge was reported from Nigeria (98.6%) [78]. This review has clearly indicated that there is a wide range of possible differences between contexts comparing to the scoping review done in SSA, which has reported the variation was between 42%-53% [50]. This difference might be explained in the variation in the literacy level among men in the two countries.

The pooled estimate for male partners who identified health facility as the place of delivery for the baby was 57.2%. This indicates that health systems in LMICs need to promote men’s uptake of quality antenatal care service [105, 116]. The highest and the lowest practice of identification of health facility as a place of birth for the baby were reported from SSA. Men in Tanzania showed poor involvement in identifying a health facility (1.8%) [99], while men in Ethiopia participated actively to identify health institutions for the birth of the baby 95.7% [92].

The pooled magnitude of male partners who accompanied their wife/partner to antenatal care follow up was 45.7%. Studies conducted in different parts of Ethiopia reported both the lowest (9.9%) [68] and the highest (88.5%) [94] levels of male partners who visited antenatal clinic with their wife/partner for pregnancy checkup.

Policymakers and program planners have to make targeted interventions by reviewing maternal and neonatal healthcare delivery guidelines to include context-specific evidence and develop evidence-informing interventions promoting male partner’s active involvement in birth preparedness and complication readiness.

### Strengths and limitations of the study

This systematic review and meta-analysis revealed the magnitude of BPCR among male partners of pregnant women and nursing mothers in LMICs as updated evidence. Stringently applying the PRISMA guideline and the Joanna Briggs Institute Meta-Analysis of Statistical Assessment and Review Instrument (JBI-MAStARI) during critical appraisal was a further strength to this systematic review and meta-analysis. Restricting the search strategy to literature published in English language is the limitation of this review.

## Conclusion

Previous evidence has underscored the role of the male partners in improving MNCH in low- and middle-income countries. Therefore, reviews that investigate key aspects of maternal health services such as BPCR and provide comparison across LMIC settings are critical for cross-national knowledge mobilization and learning. This study has included representative quality studies from across LMIC’s. In this study, a low proportion of male partners participated in BPCR in LMICs. However, the proportion ranged from 6 to 86%. This variation across LMIC regions requires a closer examination of the reasons for the high achieving settings, which have the potential to illuminate a new insight for policymakers.

The low proportion of male partners involvement in BPCR in this study calls for action for countries in low- and middle-income setting to review their health care policies, remove the barriers and promote facilitators to male partner’s involvement in BPCR. These could be achieved through behavioural interventions targeting male partner’s awareness, positive role-modelling, male community health workers and other tested interventions which improve male engagement. Health systems in LMICs must design and innovate scalable strategies suitable to their context to improve male partner’s practice of arrangements for a potential blood donor and transportation for complications that could arise during pregnancy or postpartum haemorrhage. Further, large scale systematic reviews and meta-analysis that addresses the various factors of hierarchical societal arrangements at the individual, filial, social, political, and economic levels are needed to facilitate understanding of the gendered aspects of maternal health care services.

## Supplementary Information

**Additional file 1.**

## Data Availability

Data will be available up on request of the corresponding author.

## Abbreviations

ANC: Antenatal Care
BPCR: Birth Preparedness and Complication Readiness
EmONC: Emergency Obstetric and Newborn Care
JBI: The Joanna Briggs Institute
JBI – MAStARI: The Joanna Briggs Institute Meta-Analysis of Statistical Assessment and Review Instrument
LMICs: Low- and Middle-Income Countries
PRISMA: Preferred Reporting Items for Systematic Reviews and Meta-analyses
PROSPERO: International Prospective Registry of Systematic Reviews
SBA: Skilled Birth Attendant
SDG: Sustainable Development Goal
SSA: Sub-Saharan Africa
