# Supplementary material for: Male partners’ participation in birth preparedness and complication readiness in low- and middle-income countries: a systematic review and meta-analysis
Source: BMC Pregnancy Childbirth. 2021 Aug 14;21:556. doi: 10.1186/s12884-021-03994-0 (PMC8364032; doi:10.1186/s12884-021-03994-0)
Supplement: Supplementary file 1 — Additional file 1. [file 12884_2021_3994_MOESM1_ESM.docx]

**PubMed (PMC) Query total of 1334 documents retrieved**

**((((((((((((((((((((("Spouses"[MeSH Terms]) OR "Male"[MeSH Terms]) OR "Patient Participation"[MeSH Terms]) OR "Social Participation"[MeSH Terms]) OR "Stakeholder Participation"[MeSH Terms]) AND "Parturition"[MeSH Terms]) OR "delivery, obstetric"[MeSH Terms]) OR "Pregnancy Complications"[MeSH Terms]) OR "Obstetric Labor Complications"[MeSH Terms]) OR "Delivery of Health Care"[MeSH Terms]) OR "delivery of health care, integrated"[MeSH Terms]) OR "Midwifery"[MeSH Terms]) OR "Financial Support"[MeSH Terms]) OR "Medical Savings Accounts"[MeSH Terms]) OR "Health Care Costs"[MeSH Terms]) OR "Transportation"[MeSH Terms]) OR "Transportation of Patients"[MeSH Terms]) OR "Blood Donors"[MeSH Terms]) OR "Health Facilities"[MeSH Terms]) AND ( "2004/01/01"[PDat] : "2020/12/31"[PDat] ))) AND (((((((((((male involvement*[tw]) OR male engagement*[tw]) OR male participation*[tw]) OR spouses engagement*[tw]) OR spouses participation*[tw]) OR husband involvement*[tw]) OR husband participation*[tw]) OR husbands' participation*[tw])) AND (((((((((birth preparedness*[tw]) OR parturition*[tw]) OR Delivery, Obstetric*[tw])) OR (((complication readiness*[tw]) OR Pregnancy Complications*[tw]) OR Obstetric Labor Complications*[tw])) OR ((((((((((Delivery of Health Care*[tw]) OR Delivery of Health Care, Integrated*[tw]) OR Midwifery*[tw]) OR Financial Support*[tw]) OR Medical Savings Accounts*[tw]) OR Health Care Costs*[tw]) OR Transportation*[tw]) OR Transportation of Patients*[tw]) OR Blood Donors*[tw]) OR Health Facilities*[tw]))) OR (((((((((identified health facility*[tw]) OR identified skilled birth attendant*[tw]) OR identified blood donor*[tw]) OR saved money for delivery*[tw]) OR arranged transport*[tw]) OR discussed place of delivery*[tw]) OR discussed who should perform delivery*[tw]) OR knowledge about danger signs*[tw]) AND ( "2004/01/01"[PDat] : "2020/12/31"[PDat] ))) AND ( "2004/01/01"[PDat] : "2020/12/31"[PDat] ))) AND ( "2004/01/01"[PDat] : "2020/12/31"[PDat] ))**

**SCOPUS Query**

**127 document results (126 articles + 1 gray literature)**

**( TITLE-ABS-KEY ( "spouses involvement" ) OR TITLE-ABS-KEY ( "spouses participation" ) OR TITLE-ABS-KEY ( "spouses engagement" ) OR TITLE-ABS-KEY ( "male involvement" ) OR TITLE-ABS-KEY ( "male participation" ) OR TITLE-ABS-KEY ( "male engagement" ) OR TITLE-ABS-KEY ( "husband involvement" ) OR TITLE-ABS-KEY ( "husband participation" ) OR TITLE-ABS-KEY ( "husbands' engagement" ) AND TITLE-ABS-KEY ( "birth preparedness" ) OR TITLE-ABS-KEY ( "parturition" ) OR TITLE-ABS-KEY ( "Delivery, Obstetric" ) OR TITLE-ABS-KEY ( "Pregnancy Complications" ) OR TITLE-ABS-KEY ( "Obstetric Labor Complications" ) OR TITLE-ABS-KEY ( "identified blood donor" ) OR TITLE-ABS-KEY ( "identified skilled birth attendant" ) OR TITLE-ABS-KEY ( "arranged transportation" ) OR TITLE-ABS-KEY ( "saved money for delivery" ) OR TITLE-ABS-KEY ( "financial support for pregnancy" ) OR TITLE-ABS-KEY ( "identified health facility" ) ) AND ( EXCLUDE ( PUBYEAR , 2002 ) OR EXCLUDE ( PUBYEAR , 1991 ) OR EXCLUDE ( PUBYEAR , 1988 ) OR EXCLUDE ( PUBYEAR , 1987 ) )**

**CINHAL QUERY total 284 articles retrieved**

TI "male involvement*" OR TI "husband participation*" OR TI "couples engagement*" AND TI "birth preparedness*" OR TI "parturition*" OR TI "complication readiness*" OR TI ( "birth preparedness and complication readiness*" ) OR TI "identified skilled birth attendant*" OR TI "identified blood donor*" OR TI "arranged transportation*" OR TI "saved money for delivery*" OR TI "identified health facility*"

Limiters - Published Date: 20040101-20211231

**Embase Query 95 document results**

**#27** #26 AND (**2004**:py OR **2021**:py) AND **'human'**/de [95](https://www-embase-com.ludwig.lub.lu.se/)

**#26** #24 AND #25 [6,071](https://www-embase-com.ludwig.lub.lu.se/)

**#25** #11 OR #12 OR #13 OR #14 OR #15 OR #16 OR #17 OR #18 OR #19 OR #20 OR #21 OR #22 OR #23 [804,542](https://www-embase-com.ludwig.lub.lu.se/)

**#24** #1 OR #2 OR #3 OR #4 OR #5 OR #6 OR #7 OR #8 OR #9 OR #10 [369,230](https://www-embase-com.ludwig.lub.lu.se/)

**#23 'pregnancy complication preparedness'** OR ((**'pregnancy'**/exp OR **pregnancy**) AND (**'complication'**/exp OR **complication**) AND (**'preparedness'**/exp OR **preparedness**)) [204](https://www-embase-com.ludwig.lub.lu.se/)

**#22 'pregnancy complication readiness'** OR ((**'pregnancy'**/exp OR **pregnancy**) AND (**'complication'**/exp OR **complication**) AND (**'readiness'**/exp OR **readiness**)) [204](https://www-embase-com.ludwig.lub.lu.se/)

**#21 'financial support for the pregnant women'** OR (**financial** AND (**'support'**/exp OR **support**) AND **for** AND **the** AND **pregnant** AND (**'women'**/exp OR **women**)) [425](https://www-embase-com.ludwig.lub.lu.se/)

**#20 'identified health facility'** OR (**identified** AND (**'health'**/exp OR **health**) AND **facility**) [17,590](https://www-embase-com.ludwig.lub.lu.se/)

**#19 'identified blood donor'** OR (**identified** AND (**'blood'**/exp OR **blood**) AND (**'donor'**/exp OR **donor**)) [18,854](https://www-embase-com.ludwig.lub.lu.se/)

**#18 'arranged transport'** OR (**arranged** AND (**'transport'**/exp OR **transport**)) [1,475](https://www-embase-com.ludwig.lub.lu.se/)

**#17 'saved money for delivery'** OR (**saved** AND (**'money'**/exp OR **money**) AND **for** AND (**'delivery'**/exp OR **delivery**)) [74](https://www-embase-com.ludwig.lub.lu.se/)

**#16 'identified skilled birth attendant'** OR (**identified** AND **skilled** AND (**'birth'**/exp OR **birth**) AND **attendant**) [103](https://www-embase-com.ludwig.lub.lu.se/)

**#15 'labor complication'**/exp OR **'labor complication'** [212,719](https://www-embase-com.ludwig.lub.lu.se/)

**#14 'pregnancy complication'**/exp OR **'pregnancy complication'** [155,235](https://www-embase-com.ludwig.lub.lu.se/)

**#13 'delivery, obstetrics'** OR (**delivery,** AND (**'obstetrics'**/exp OR **obstetrics**)) [96,232](https://www-embase-com.ludwig.lub.lu.se/)

**#12 'birth preparedness and complication readiness'**/exp OR **'birth preparedness and complication readiness'**[120](https://www-embase-com.ludwig.lub.lu.se/)

**#11 'birth'**/exp OR **birth** [490,583](https://www-embase-com.ludwig.lub.lu.se/)

**#10 'couples participation'** OR ((**'couples'**/exp OR **couples**) AND (**'participation'**/exp OR **participation**)) [841](https://www-embase-com.ludwig.lub.lu.se/)

**#9 'couples engagement'** OR ((**'couples'**/exp OR **couples**) AND (**'engagement'**/exp OR **engagement**)) [446](https://www-embase-com.ludwig.lub.lu.se/)

**#8 'couples involvement'** OR ((**'couples'**/exp OR **couples**) AND (**'involvement'**/exp OR **involvement**)) [955](https://www-embase-com.ludwig.lub.lu.se/)

**#7 'husband participation'** OR ((**'husband'**/exp OR **husband**) AND (**'participation'**/exp OR **participation**)) [312](https://www-embase-com.ludwig.lub.lu.se/)

**#6 'husband engagement'** OR ((**'husband'**/exp OR **husband**) AND (**'engagement'**/exp OR **engagement**)) [72](https://www-embase-com.ludwig.lub.lu.se/)

**#5 'husband involvement'** OR ((**'husband'**/exp OR **husband**) AND (**'involvement'**/exp OR **involvement**)) [376](https://www-embase-com.ludwig.lub.lu.se/)

**#4 'male participation'** OR ((**'male'**/exp OR **male**) AND (**'participation'**/exp OR **participation**)) [89,208](https://www-embase-com.ludwig.lub.lu.se/)

**#3 'male engagement'** OR ((**'male'**/exp OR **male**) AND (**'engagement'**/exp OR **engagement**)) [37,915](https://www-embase-com.ludwig.lub.lu.se/)

**#2 'male involvement'** OR ((**'male'**/exp OR **male**) AND (**'involvement'**/exp OR **involvement**)) [254,502](https://www-embase-com.ludwig.lub.lu.se/)

**#1 'male involvement'** OR ((**'male'**/exp OR **male**) AND (**'involvement'**/exp OR **involvement**)) [254,502](https://www-embase-com.ludwig.lub.lu.se/)
